# Correction to: S08-3: Goals and realities in local governments’ involvement in PA promotion for diverse populations subgroups – a comparative analysis of municipalities from 5 countries

**DOI:** 10.1093/eurpub/ckaf134

**Published:** 2025-08-19

This is a correction to: Petru Sandu, Peter Gelius, Sven Messing, Antoine Noel-Racine, Yuko Oguma, Tanja Onatsu, Antonia Paula Papiu, Yoshinobu Saito, Noriko Takeda, Katariina Tuunanen, Anne Vuillemin, Razvan Mircea Chereches, S08-3: Goals and realities in local governments’ involvement in PA promotion for diverse populations subgroups – a comparative analysis of municipalities from 5 countries, *European Journal of Public Health*, Volume 34, Issue Supplement_2, September 2024, https://doi.org/10.1093/eurpub/ckae114.234

Originally, the incorrect article was published with this author list and title. This is now emended, and the correct article is published to the author list and title. Additionally, there was a typographical error in the title. This is now emended to read: “governments” instead of: “goverments”.

Addendum published on 16 October 2025: this is an addendum to the above correction notice. The title of the article above the correction notice was originally published with a typographical error - this should read: “[...from 5 countries” instead of “[…] from 5 countrie

